# Quality of Poultry Roast Enriched with Hemp Seeds, Hemp Oil, and Hemp Flour

**DOI:** 10.3390/foods11233907

**Published:** 2022-12-03

**Authors:** Anna Augustyńska-Prejsnar, Jadwiga Topczewska, Małgorzata Ormian, Zofia Sokołowicz

**Affiliations:** Department of Animal Production and Poultry Products Evaluation, Institute of Food Technology and Nutrition, College of Natural Science, University of Rzeszow, 35-959 Rzeszow, Poland

**Keywords:** meat product, nutritional value, physical and chemical parameters, sensory properties, hemp products

## Abstract

The aim of this study was to add natural hemp components to poultry roast recipes, to enhance their quality (physical, chemical, and sensory qualities of the product). Two variants of poultry roast (group P_1_ and P_2_) with a 10.2% addition of hemp components and a traditional equivalent with the participation of animal fat (group K) were tested. In the roast of group P_1_, the share of hemp seeds was 8%, hemp flour 0.2%, and hemp oil 2%; while in group P_2_, the proportions were 4%, 0.2%, and 6%, respectively. Roasts with hemp components were found to be characterized by a darker color; lower cooking losses; higher fiber content, and lower cholesterol and fat content; a favorable fatty acid ratio PUFA; n-3 and n-6; and acceptable sensory characteristics compared to the control group. Products with a higher (8%) share of hemp seeds contained more protein and fiber and were characterized by a higher degree of yellow saturation (b*), lower cooking losses after heat treatment, and a higher desirability of taste and better binding. Products in group P_2_, with a higher (6%) hemp oil content, had a lower cholesterol content and a lower proportion of SFA fatty acids and a higher proportion of omega-3 fatty acids, but were assessed as rated lower in terms of taste and binding.

## 1. Introduction

Increasing consumer awareness of food quality, production trends, the relationship between diet and health, and lack of time are the most common reasons for introducing new, more nutritional products to the market, as well as easy-to-prepare meat products [1,2,3]. The addition of natural bioactive compounds to meat products is nowadays a popular trend in meat processing [4,5,6]. Currently, enriched meat products are obtained thanks to modification at the raw material production stage (appropriate selection of animal breed, in terms of the animal raw materials obtained) and selection and supplementation of feed [6,7], and enrichment of meat products at the processing stage. The enrichment of products is based on the introduction of natural non-meat ingredients into meat products, in order to increase the nutritional and health value of the product or to improve the product in terms of the content of a given ingredient. The most common natural ingredients are plant proteins, fiber, vitamins, and minerals, as well as herbs and seeds of plants rich in bioactive ingredients with proven health-promoting properties [3,5,6]. The health values of meat products can also be improved by using probiotics, reducing calorific value or cholesterol levels, as well as removing non-nutrients [8]. Of particular interest to consumers are foods with increased levels of essential fatty acids, which are beneficial to human health. Vegetable fats are increasingly common substitutes for animal fat. As indicated by recent studies [9,10,11,12,13,14,15], the addition of vegetable oils, including, for example, flaxseed oil, chia oil, hemp oil, canola oil, olive oil, sunflower oil, tiger nut oil, algae oil, or the use of vegetable oils as a substitute for animal fat in meat products meets the nutritional demand for enriching such products with unsaturated fatty acids (n-3, n-6, and n-9). Excessive consumption of animal products with large amounts of fat leads to obesity, and increases the risk of cardiovascular disease and the risk of developing certain cancers, such as of the colon [9]. In addition, consumers of meat preparations seek products containing natural ingredients, and insist that the use of synthetic additives be minimized or discontinued [2].

For several years, there has been an increase in interest in the properties of hemp (*Camnabis sativa* L.). Hemp seeds are widely used in the food industry and are considered to be one of the most nutritionally complete food sources [16]. They can be consumed as whole and shelled seeds, as well as in the form of processed products, including oil, flour, and protein powder [17,18]. Hemp seeds are a rich source of fat (25–35%), protein, fiber, calcium, iron, phosphorus, zinc, and magnesium. The presence of edestine, phytic acid, choline, lecithin, trigonelline, vitamin K, and tocopherols in hemp seeds is one of their characteristic features. Terpene hydrocarbons are responsible for the taste and smell of hemp, mainly β-caryophyllene and α-humulene (sesquiterpenes), as well as monoterpene and mycene [19]. Hemp protein is characterized by high digestibility and good composition of amino acids, thanks to which it can be effectively used by the human body and is a source of bioactive peptides [20,21]. Cold-pressed hemp seed oil is also of great interest to the food industry. Hemp oil contains more than 80% unsaturated fatty acids, including linoleic acids (50–70%), α-linolenic (15–25%), and γ-linolenic (3–5%), with an optimal balance of polyunsaturated omega-6 to omega-3 fatty acids, in a ratio of 3:1. It is rich in phytosterols, tocopherols, polyphenols, carotenoids, phospholipids, cannabidiol (CBD), and P-sitosterol, as well as being characterized by anti-inflammatory, antibacterial, and antioxidant properties [17,22,23,24]. Hemp flour is a product derived from hemp seeds after dehulling, grinding and defatting at low temperatures. It is a product with high nutritional value. It contains approximately 30% easily digested protein, with a high edestine and albumin content, as well as comprising 5.6% fats, rich in unsaturated fatty acids [25]. It is distinguished by its functional properties, due to its high content of phenolic compounds (ferulic acid, caffeic acid, chlorophyll, and carotenoids) with anticancer and antioxidant properties. It also contains vitamins E, A, K_1_, D_2_, B_1_, B_2_, and B_3_, coenzyme Q10, and microelements [19,26].

Poultry roast is a processed meat product. In the development of recipes for processed poultry products, the proportion of poultry meat should be a minimum of 50%, and recipe formulation requires the use of standardized raw materials and other ingredients of good quality. Turkey meat obtained from the thigh can be a valuable raw material for the production of processed products, due to its high nutritional value (high content of biologically valuable protein, favorable fatty acid profile, low calorific value), favorable sensory characteristics, and high suitability for processing [27,28,29].

The aim of this study was to add natural hemp components (hemp seed, hemp oil, hemp flour) to poultry roast recipes, to enhance their quality (physical, chemical, and sensory qualities of the product). This study will increase knowledge on the possibilities for the use of hemp products in the meat industry and facilitate the development of alternative meat products with high nutritional and health value.

## 2. Materials and Methods

### 2.1. Materials and Components

Prior to the first series of tests, the raw animal material (turkey drumstick meat—45 kg and pork jowl—5 kg) from the same producers were purchased at the same time at the wholesale store Makro Cash & Carry, Rzeszow, Poland (to eliminate the effect of variation). The raw material was then divided into three equal portions (with a weight of 0.5 kg), sealed in vacuum packs using an MINI 171178-4160AP (QK-IT, Spilimbergo, Italy) vacuum packaging machine, and stored frozen at −20 °C ± 2 °C for three weeks in a GN 3056 Premium freezer (Liebherr, Ulm, Germany). The chemical composition of the raw turkey drumstick meat was as follows (%): total ash 1.09, protein 22.10, lysine 1.77, methionine 0.58, cysteine 0.24, tryptophan 0.23, fat 4.18, n-6 fatty acids: 25.60, and n-3 2.06. The chemical composition of the raw pork jowl was (%): total ash 0.53, protein 10.40, lysine 0.53, methionine 0.58, cysteine 0.90, tryptophan 0.08, fat 49.70, n-6 fatty acids 9.00, and n-3 0.62 The plant ingredients used were hemp seed without husk and hemp flour from the company Bio Planet, certified as an organically grown product PL-EKO-07, and cold-pressed hemp oil from the company BioKonopia, sourced from organically grown seeds CZBIO 002, purchased from an organic food shop. The hemp seeds featured (%): total ash 5.17, protein 32.50, lysine 1.07, methionine 0.76, cysteine 0.51, tryptophan 0.36, fat 53.09, the fatty acids n-6 56.90, and n-3 17.40. According to the manufacturer’s declaration the hemp flour featured total ash 7.45%, protein 31.70%, and fat 5.62%; the hemp oil featured protein 1.0% and fat 94.0%, including saturated fatty acids 10%, monounsaturated fatty acids 15.0%, and polyunsaturated fatty acids 75.0%, including n-6 54.0% and n-3 18.0%.

### 2.2. Preparation of the Poultry Roast

Three experimental series were performed, in which three variants of poultry roast were produced, according to the same production scheme. The recipe composition is presented in Table 1. The control group (K) consisted of turkey meat roast with the addition of pork jowl. In the experimental groups, the pork jowl was replaced with hemp products. In the P_1_ group roast, the share of hemp seeds was 8%, hemp flour 0.2%, and hemp oil 2%; while in group P_2_, the proportions were 4%, 0.2%, and 6%, respectively. In our study, hemp products were used in three forms: hemp seeds, hemp oil, and hemp flour. The selection of the proportions of hemp products was based on theoretical calculations preceded by studies of the chemical composition of animal and vegetable raw materials used in the production of finished products, which allowed obtaining products of a health-promoting nature (favorable composition and desired nutritional value). Previous pilot studies produced products with acceptable taste and sensory characteristics, since hemp flour has a characteristic bitter taste. In each batch, a portion of turkey meat and jowl was thawed at 4 °C in an FKv36/10 refrigeration cabinet (Liebherr, Ulm, Germany), until a temperature of 4 °C ± 1 °C was reached inside the raw material, before the poultry roast was processed. The turkey meat was minced in a meat grinder (Zelmer, Rzeszów, Poland) equipped with a cutting set with a mesh with holes of 4 mm diameter, weighed to the nearest 1 g, and divided into three parts (5 kg each), while the pork jowl was minced with a mesh of 2 mm diameter (0.5 kg). The hemp seed was ground (Hallde Cutter VCB-32 Stockholm, Sweden) to a particle fraction of 0.02 ≤ Φ≤ 2.00 mm, then heated to 90 °C, and cooled to 20 °C ± 2 °C. In each group, all ingredients were introduced into a mechanical meat mixer with a stainless steel stirrer (Titanium Kenwood, Havant, UK) and stirred for 5 min, until the ingredients were evenly distributed. The resulting stuffing was placed in 250 g aluminum molds and fixed by baking in a convection-steam oven with an integrated thermo-probe (4in1 SOUS VIDE AEG BSK792320M Berlin, Germany) at 180 °C, relative humidity of air 10 °C, and thermoelectric off, until the geometric center of the product reached 76 °C ± 2. The interior temperature of the product was monitored in real time. The roasts were preliminarily cooled at 20 °C and then cooled in a FKv36/10 cooling cabinet (Liebherr, Ulm, Germany) at 4 °C for 0.5 h. For the study, 45 kg of turkey drumstick meat was purchased. Three series of tests were performed, for this purpose the meat was divided into three groups of 15 kg each, which represented 6–6.5 shanks (the weight of turkey shanks ranged from 800 to 950 g). In each series of tests, the raw meat material (15 kg) was ground and the finished products were prepared. Specific tests (physical, chemical, and sensory characteristics) were performed on the finished products, which were performed for each variant of the product, as described in the methodology. Six samples were randomly taken from the mass of the ground muscle in each series of tests to determine the chemical composition of the meat.

### 2.3. Determination of Quality Features

#### 2.3.1. Physical Characteristics

The pH of the products was determined using an HI 99,163 digital meter (Hanna Instrument Company, Vöhringen, Germany) by inserting a spear-type combination electrode directly into the product. Before each measurement, the device was calibrated using pH 4.0 and pH 7.0 buffer solutions. The mean pH value was determined from 10 measured values for the same product, and the procedures were the same for all samples [30]. The cooking losses were calculated by weighing the samples before and after thermal treatment, according to the formula: weight before roasting—weight after roasting/weight before roasting/× 100. Cooking losses after heat treatment were calculated as the difference in the masses of the samples before and after heat treatment, which were expressed as a percentage. The color of the cross-sectional area of the poultry roast was measured using a Chroma Meter colorimeter (Konica Minolta, Osaka, Japan), equipped with a CR 400 head (ø = 11 mm). The reflection method was used with standard lighting: D_65_ and observer angle 2°. The device was calibrated according to the manufacturer’s recommendations. The CIE values of L* (lightness), a* (redness), and b* (yellowness), according to the color systems of the International Commission on Illumination [31], were determined from the average of three randomly selected readings of the cut surface of each sample. Each reading for a point was repeated 3 times, and the final value for each sample was the average of these readings [32]. Tenderness was measured based on the cutting force (Fmax), using a BT1-FR1.OTH.D14 Zwick/Roelltesting machine (from ZwickCmbH & Co.KG., Ulm, Germany), applying a wide-width Warner-Bratzler (V-blade) with a head speed of 100 mm·min^−1^, and a 0.2 N pre-cut force [33]. Cutting was done on chilled samples (4 °C ± 1 °C) cut from the center of the roast with a cross section of 200 mm^2^ and a length of 50 mm [30,34].

#### 2.3.2. Chemical Characteristics

Assays were performed in triplicate. For the determination of total ash, the samples were incinerated at 525 ÷ 900 °C and the ignition residue (PB-19/LF) was determined by weight. For the determination of the protein content, mineralization of the sample was carried out in the presence of a catalyst of the organic nitrogen compounds contained in it in ammonium sulfate (VI), using a concentrated solution of sulfuric acid [32]. After alkalizing the mixture, the resulting ammonia was distilled with steam and bound in a solution of boric acid. The distillate was then titrated with a standard solution of hydrochloric acid using the distillation–titration method (Kjeldatherm mineralisation block, Gerhardt, Königswinter, Germany, with controlled temperature control, Vapodest Carousel automatic distiller, Gerhardt, Königswinter, Germany). The calculated amount of nitrogen was converted into protein by multiplying by a conversion factor of 6.25. For the determination of total fat, hydrolysis of the sample with a hydrochloric acid solution was carried out, to release the fat from the protein–fat and sugar–fat complexes, then the fat was separated from the hydrolysate by filtration through a filter. After fat extraction in a Soxhlet apparatus (Soxtherm, Gerhardt, Königswinter), the fat content of the sample was determined by weight. Fiber was determined by the enzymatic-weight method in the sample in two parallel repeats. The sample was treated with alpha-amylase to decompose starch, followed by protease and amyloglucosidase, to break down proteins and the remaining starch, respectively. The soluble dietary fiber was precipitated with an ethanol solution and separated by filtration and dried, and subsequently the fiber content was determined (Kjeldatherm mineralisation block, Gerhardt, Königswinter, Germany with controlled temperature control, Vapodest Carousel automatic distiller from Gerhardt, Germany). Vitamin E was designated as the DL-alpha-tocopherol content. Tocopherol was released from the sample matrix by ethanol extraction with simultaneous hydrolysis of its esters. The tocopherol content in the extract was determined by high-performance liquid chromatography with a fluorescent detector. Regarding the total amino acids, these are bound in proteins, so the peptide bonds must be broken, to free the amino acids for analysis. The procedure we followed with acid hydrolysis broke the peptide bonds. For the hydrolysis, we prepared our samples using 6 M Hydrochloric acid (HCl) and an internal standard of 0.5 M Norvaline. After 20–24 h at 110 °C, we diluted it with water in a 100 mL flask. Then we filtered and performed the derivatization with a Waters kit. In order to quantify amino acids, we used calibration curves with standards that contained known quantities. The method for determining the fatty acid profile involved the preparation of fatty acid methyl esters. For the preparation of fatty acid methyl esters, a method using boron trifluoride and extraction of the obtained esters with isooctane was used. The resulting mixture of esters was separated on a chromatographic column and detected using a GC-FID detector. Cholesterol determination was performed by gas chromatography with FID detection using a GC 7820A gas chromatograph (Agilent Technologies, Inc., Agilent, Santa Clara, CA, USA). The measurements were carried out in a laboratory with controlled temperature conditions. Sterols were isolated from the matrix, and then the samples were derivatized using gas chromatography with flame-ionization detection (FID). All chemical analyses were performed in an accredited GBA Polska laboratory.

### 2.4. Assessment of Sensory Qualities

The sensory evaluation of poultry roast samples was carried out by a 10 member team, which included women aged 26–65; an assessment team with proven sensory sensitivity and at least 4 years of experience in carrying out sensory assessments using the scaling method. The study used special assessment cards to describe the sensory properties of the samples. In order to properly evaluate the samples, the roast was cut into slices measuring 2 cm × 2 cm × 4 cm, fitted with a code, and presented to the evaluators in white containers. The samples were evaluated randomly, in three repetitions. The temperature of the samples during sensory evaluation was 20 °C ± 1 °C. Between each sample test, the evaluators took a 30 s break and rinsed their mouths with mineral water. A 5 point hedonic scale was used. The attributes of the assessment were: intensity of smell and taste, desirability of smell and taste, juiciness, tenderness, bonding, consistency, and general acceptability [35]. The test was carried out in a suitably prepared room, free from foreign odors, at a temperature of 20 °C, and with lighting, eliminating any distractions, in accordance with the applicable standards [36].

### 2.5. Statistical Analysis of Data

The research results are tabulated as mean values and standard deviations. The impacts of enrichment of poultry roast with hemp components (hemp seeds, hemp oil, and hemp flour) on the physical and chemical characteristics were assessed with one-way ANOVA (using post hoc tests and Tukey’s HSD) and on the sensory characteristics using non-parametric Kruksal–Wallis tests. Differences were considered to be significant at *p* < 0.05. The calculations were performed using the Statistica 13.3 software package [37].

## 3. Results and Discussion

The acidity of a meat product is a quality parameter related to its technological and sensory properties and safety [5]. Regardless of the amount of ingredients used within the study groups, the pH remained virtually unchanged (*p* > 0.05), and the pH values of the analyzed samples ranged from 6.40 to 6.41 (Table 2). Similar results were obtained by Kotecka-Majcharzak et al. [21] in pork meatballs with a varied amount of hemp cake and by Bozhko et al. [38]. In turn, Kerner et al. [5] showed the higher pH of pork burgers with the addition of dried and pressed hemp cake and sweet grass.

Cooking losses from heat treatment are perceived by consumers as undesirable effects, reducing the acceptance of meat products [10]. Our own study showed that the addition of hemp products in both group P_1_ and P_2_ significantly (*p* < 0.05) reduced the product cooking losses after heat treatment compared to the control group (Table 2). Within the study groups, products from group P_1_, with a predominance of hemp seeds (8%), were characterized by lower cooking losses, which may be important from a technological point of view. Smaller cooking losses during roasting (release of water and fat) can be attributed to the fiber present in hemp seeds and flour. Reduction of thermal losses in pork loaves with the addition of hemp ingredients (hemp protein, hemp flour, and hemp seeds) was shown by Zając et al. [20]. However, the extent of the losses was determined, not only by the share, but also by the form of the ingredients used. Kotecka-Majrzak et al. [21] showed that with an increase in the ratio of the hemp additive used to the meat product, losses after cooking decreased. In a study by Sun et al. [39], the addition of hydrated hemp seed flour significantly reduced the culinary losses of poultry sausages, and in a study by Kerner et al. [5], the addition of hemp cake made it possible to reduce losses when grilling pork burgers. The results of a study by Yuan et al. [40] showed that micronized cold-pressed hemp cake can be used as a phosphate replacement to reduce losses during the cooking of frankfurters. In turn, in a study by Botella-Martinez et al. [10], replacing pork bacon with hemp oil in beef burgers resulted in a greater culinary quality loss.

The inclusion of natural hemp ingredients in the recipe in the studied groups (P_1_, and P_2_) had a significant (*p* < 0.05) effect on the color of the poultry roast cross-section. Meat products with hemp ingredients were darker in color, with lower values of L* (lightness), a* (redness) and b* (yellowness) compared to the control group (Table 2). Within the study groups, roasts with a higher (6%) proportion of hemp oil (group P_2_) were characterized by a lower brightness index. The changes in color were due to the significant proportion of hemp products (10.2%) containing chlorophyll. Similar results were obtained by Sun et al. [39], after incorporating hemp flour into pork sausages. In a study by Zając et al. [20], it was shown that the color parameters of pork loaves changed depending on the hemp ingredient used, with a tendency toward darkening of the color, including a decrease in the red parameter a*. Kotecka-Majchrzak et al. [21] showed that with a higher share of hemp cake, the color of the cross-section of meat meatballs changed towards a darker one. In a study by Zając and Świątek [41], instrumental analysis of color did not show the effect of the addition of hemp seeds and flax on the color changes in meat pâtés.

In the assessment of the instrumental texture of meat products, the most commonly used parameter related to tenderness is the value of the maximum cutting force [32]. In our own research, it was shown that roasts enriched with hemp products were characterized by the (*p* < 0.05) significantly greater force necessary to cut with a Warner-Bratzler flat-bladed knife. The difference from the control group was 4.40 N in group P_1_ and 3.56 N in group P_2_. The increase in hardness measured by the TPA test of meat products enriched with plant additives (hemp and flax seeds) was confirmed in a study by Zając and Świątek [41], with hemp ingredients (seeds, flour, protein) by Zając et al. [20], and with hemp cake by Kotecka-Majchrzak et al. [21]. The authors showed that the hardness of the products increased with the increase in the hemp additive used. Studies by Merenkov et al. [42] indicated that the addition of hemp protein containing proteins of a globular nature improved the stability of the rheological characteristics of minced meat. Different results were obtained by Sun et al. [39] in boiled poultry sausages with the addition of hemp flour, which resulted from the previous hydration of the additive used.

Proteins are an essential component of the human diet, and their supply via food determines the proper functioning of the body. Protein deficiency in the diet leads to inhibition of growth and development of the body, prevents tissue restoration, and reduces resistance to diseases [43]. In our study, it was shown that enrichment of the meat product with hemp products had a beneficial effect on increasing the protein content (*p* < 0.05) in group P_1_, with a higher content of hemp seeds compared to group P_2_ and the control group (Table 3). Similar results have also been shown by other authors: Zając et al. [20], Bozhko et al. [38], Augustyńska-Prejsnar et al. [32], and Zinina et al. [18], who used high-protein hemp products (hulled hemp seeds, hemp flour, and hemp protein) to enrich meat products. In contrast, Kerner et al. [5] did not observe an increase in protein in a pork burger after the introduction of 2% hemp cake. In studies by Sun et al. [39], adding hydrated hemp flour to poultry sausage significantly reduced the protein and fat content, but increased the total ash and fiber content. Vegetable proteins are relatively low in essential amino acids compared to proteins of animal origin [18]. In our research, the introduction of hemp components into the recipe composition did not significantly change the amino acid profile of the final products (Table 3). A significant reduction (*p* < 0.05) in lysine and tyrosine content was noted in P_2_, containing a higher hemp oil content (6%), compared to P_1_, with a higher proportion of hemp seeds (8%), and the control product. An increase in the share of aspartic acid and arginine was only shown in group P_1_ (Table 3), which can be considered beneficial for improving the qualities of roasts enriched with hemp components. According to the results of the study by Zinina et al. [12], the use of a balanced combination of different vegetable protein ingredients for ground beef can provide a high-quality product. Van Vilet et al. [44] noted that consuming several sources of protein can provide a balanced amino acid profile in food.

Dietary fiber, although not a nutrient, has a significant impact on human health, including a longer-lasting feeling of satiety; promotion of the development of beneficial intestinal microflora for the body; support for the maintenance of proper bowel function and preventing constipation; support for proper intestinal peristalsis, protecting against the development of diverticula and colorectal cancer; an impact on the proper concentration of cholesterol in the blood; reduction of the concentration of glucose in the blood on an empty stomach; and compensation for the postprandial increase in its concentration [36]. Our research confirmed the possibility of increasing the fiber content in meat products through the use of hemp ingredients (Table 3). Similar results were obtained by Zając et al. [20], after adding hemp flour, hemp protein, and dehulled and whole hemp seeds to a ground pork product; as well as by Augustyńska-Prejsnar [32], using a mixture of hemp, flax, and amaranth seeds as an additive to poultry pâtés.

Among fat-soluble vitamins, a special role is played by vitamin E, the main component of which is alpha-tocopherol. Alpha-tocopherol is one of the most important compounds found in vitamin E, and it is very important, mainly due to its strong antioxidant properties and important role in the stabilization of biological membranes. Thanks to its properties, vitamin E effectively reduces the occurrence of oxidative stress and the formation of free radicals that cause aging and cell death [45,46,47]. In our study, there was no significant (*p* > 0.05) increase in vitamin E in the enriched products (Table 3). In the work of Augustyńska-Prejsnar et al. [32], the effect of the addition of a seed mixture containing 8% hemp seeds on the increase in vitamin E content in poultry pâtés was demonstrated. An increase in the content of α-tocopherol in a meat product was obtained by Dominguez et al. [48].

In our study, the modification of the poultry roast recipe resulted in a reduction in the fat content of the roasts with the addition of hemp products (Table 3). Vegetable fats are less stable than animal fats, in terms of composition, so meat products enriched with vegetable oils may contain less fat compared to their meat-fat counterparts of animal origin. This trend is not apparent in raw products [12]. According to Rodriguez-Estrada et al. [49], vegetable fats can be lost or reacted during thermal processing. Reductions in the fat content of meat products using a substitute for animal fat, most commonly lard, with vegetable fats have been confirmed in a number of research papers [9,10,11,13,14]. In a study by Barros et al. [11], a significant decrease in the fat content of hamburgers was observed with animal fat replacement in the form of tiger nut oil. Both the complete replacement of animal fat with a gelatinous emulsion of algae oil [50] and the partial replacement of pork fat with a mixture of 10% pork fat and 5% canola oil [51] resulted in a reduced total fat content in beef burgers. Partial replacement of pork fat with microencapsulated fish oil also reduced the fat content in Frankfurter sausages [48] and dry-cured sausages [52].

The fat in the enriched products was characterized by a more favorable fatty acid profile compared to the control group (Table 4). The addition of hemp products resulted in a significant reduction (*p* < 0.05) in the content of saturated fatty acids (SFA) and monounsaturated fatty acids (MUFAs), and an increase (*p* < 0.05) in the share of polyunsaturated acids (PUFAs) in the poultry roast (Table 4). The increase in the content of polyunsaturated acids and the reduction in the share of saturated acids testified to the improvement of the health-promoting qualities of poultry roasts with the inclusion of the additives. According to Markowska et al. [23], Farinon et al. [2], and Dąbrowski and Skrajda [22], hemp seed oil is characterized by a very favorable profile of fatty acids, with a low content of saturated fatty acids (SFA) and a high content of unsaturated acids (PUFAs). Reducing the proportion of saturated acids (SFA) in roasts with the inclusion of hemp additives is beneficial, because lowering the content of SFA acids in food reduces the risk of the development of atherosclerosis, embolisms, and ischemic heart disease and stroke [53]. In a study by Carvalho Baross et al. [11], replacing beef fat with tiger nut oil emulsion in a beef burger resulted in a decrease in the proportion of saturated fatty acids (SFA) and an increase in MUFAs and PUFAs. According to Botella Martinez et al. [10], partially replacing pork fat with chia and hemp oils is a strategy for obtaining healthier pork burgers, with higher PUFA and lower SFA. In addition, other authors [13,14,47,48], by changing the fatty acid profile by replacing animal fats with vegetable oils, obtained healthier products, rich in the necessary unsaturated fatty acids. In our study, the share of health-beneficial polyunsaturated fatty acids (PUFAs) in roasts enriched with hemp products was about 50% higher than in the control group, which also indicates the more beneficial health qualities of these products. PUFAs exert, among other things, a beneficial effect on reducing blood cholesterol levels [54]. The results confirmed previous studies by Botella-Martinez et al. [28] on the fatty acid profile in burgers after replacing pork fat with hemp oil with the addition of chia oil.

The human body is unable to synthesize n-3 and n-6 polyunsaturated fatty acids due to the lack of enzymatic systems capable of forming a double bond in the fatty acid chain in a position further than C-9. For this reason, polyunsaturated fatty acids must be supplied via food. In the human body, n-3 and n-6 fatty acids perform important functions, because they are part of the phospholipids of cell membranes. Their mutual proportion in tissues depends to a large extent on their supply in the diet [53,54,55]. In the analysis of the obtained research results, it is worth noting that the share of n-3 acids in the enriched products was almost five-times higher in products from group P_2_ (with a predominance of hemp oil) and four-times higher in products from group P_1_ (with a predominance of hemp seeds) compared to the control group (Table 4). Omega-3 acids perform important functions in the human body: they reduce the concentration of triglyceride levels, which are undesirable when in excess in the blood; they normalize blood pressure; they have anticoagulant and anti-inflammatory effects; they inhibit the development of coronary heart disease and coronary artery disease; they inhibit excessive immune response; they have an antiatherosclerotic effect; improve vision; improve the function of nerve cell membranes of the cerebral cortex (antidepressant effect); protect against obesity; and also have a beneficial effect on the skin [54]. Omega n-6 acids have, among other things, anti-inflammatory and immunomodeling properties, and stabilize blood pressure [53,56]. One of the indicators of the quality of a healthy human diet is, not only the level of consumption of EFAs from the omega-3 family, but also the ratio of omega-3 to omega-6 acids. Knowing the biological action of omega-3 and omega-6 acids, the correct ratio of omega-6:omega-3 supply has been determined, which is 2–5:1. In products enriched with hemp additives, the ratio of omega-3 to omega-6 was favorable and was 1:5 in group P_1_ and 1:4.5 in group P_2_, while in the control group, the ratio between omega-3 and omega-6 was 1:12. An improvement in the ratio of omega-3 to omega-6 fatty acids was also demonstrated by Zając and Świątek [41] and Zając et al. [20], after adding hemp seeds to ground meat products; and by Augustyńska-Prejsnar et al. [32], after enriching poultry pâtés with a seed mixture with hemp.

Cholesterol is a lipid compound that is necessary for the proper construction and functioning of the body. It stabilizes biological membranes and is a substrate for the synthesis of many important compounds (sex hormones, corticosteroids, vitamin D, bile acids), but its excess in the body can be harmful and may lead to an increased risk of cardiovascular diseases. In the human body, cholesterol comes from both food sources and de novo biosynthesis. The effect of diet on blood cholesterol levels in people with normal lipid management is not significant, but for people who have been found to have lipid disorders, a diet with a lower cholesterol content may have a beneficial effect on health [57]. This study indicated that the replacement of animal fat with hemp components significantly reduced the cholesterol content of products from groups P_1_ and P_2_ (Table 4).

When developing enriched meat products involving the replacement of meat or fatty raw material with vegetable components, it is necessary to obtain sensory characteristics corresponding to their traditional counterparts [38]. In the conducted study, sensory analysis showed that meat products with hemp ingredients from group P_1_ and P_2_ were characterized by higher smell desirability and better consistency compared to the control group (Table 5). However, the panelists gave the highest marks in desirability of taste and binding for poultry roasts enriched with a higher (8%) share of hemp seeds (group P_1_). In contrast, the tenderness of products with hemp inredients was rated lower than the control group with a higher proportion of animal fat. Similarly, in a study by Zając et al. [20], among all the evaluated meat products with the addition of hemp ingredients, the product enriched with dehulled hemp seeds was characterized by a higher consumer acceptability. In a study by Augustyńska-Prejsnar et al. [32], it was shown that with a 24% share of plant ingredients, the addition 8% of hemp seeds had a positive effect on the taste desirability and binding of meat and plant pâtés. According to Zając and Świątek [41], the overall sensory quality of pâtés with the addition of hemp seeds and golden flax was rated higher compared to the product without ingredients. According to Kotecka-Majchrzak [21] and Naumova et al. [8], the amount of additive used is important for the sensory assessment of a product enriched with the addition of hemp ingredients. In a work by Bozhko et al. [27], the use of 8–12% hemp flour allowed obtaining a meat and vegetable product acceptable to consumers.

## 4. Conclusions

The results of this study indicated that the addition of hemp components as a substitute for animal fat allowed poultry roasts with increased nutritional value and acceptable sensory characteristics to be obtained. The addition of hemp products in both group P_1_ and P_2_ reduced the product cooking losses after heat treatment, affected color darkening, and increased hardness measured by cutting force compared to the control group. Roasts from group P_1_ and P_2_ were characterized by a high fiber content and a favorable ratio of fatty acids and a lower fat and cholesterol content compared to the control group. Within the study groups, products with a higher (8%) share of hemp seeds contained more protein and fiber and were characterized by a higher degree of yellow saturation (b*), lower cooking losses after heat treatment, and higher desirability of taste and better binding. Products in group P_2_, with a higher (6%) hemp oil content, had a lower cholesterol content and a lower proportion of SFA fatty acids and a higher proportion of omega-3 fatty acids, but were assessed as rated lower in terms of taste and binding.

The results obtained confirm that hemp products such as seeds, hemp flour, and hemp seed oil can be used in the processing of meat as a health-promoting ingredient, provided that the level tolerated by consumers is maintained.

## Figures and Tables

**Table 1:** Recipe components of the poultry roast (%).

Ingredients		Variants of the Product		
		Group K	Group P_1_	Group P_2_
Meat from turkey broiler thighs		80.80	80.80	80.80
Pork jowl		10.20	-	-
Hemp components	Seed	-	8.00	4.00
	Flour	-	0.20	0.20
	Oil	-	2.00	6.00
Chilled water		8.00	8.00	8.00
Non-iodized salt		0.90	0.90	0.90
Black pepper		0.10	0.10	0.10

**Table 2:** Effect of enrichment with seeds, flour, and hemp oil on the active acidity, cooking losses, and physical characteristics of poultry roast (x ± s).

Studied Parameters	Poultry Roast		
	Group K	Group P_1_	Group P_2_
pH	6.45 ± 0.01	6.41 ± 0.02	6.40 ± 0.03
Cooking losses (%)	19.70 ± 1.20 ^a^	14.90 ± 1.30 ^c^	16.01 ± 1.10 ^b^
Color cross-section:			
L*—lightness	66.88 ± 0.95 ^a^	62.94 ± 1.00 ^b^	60.40 ± 2.3 ^c^
a*—redness	9.61 ± 0.26 ^a^	7.40 ± 0.51 ^b^	7.59 ± 0.62 ^b^
b*—yellowness	12.78 ± 0.37 ^a^	14.44 ± 0.25 ^b^	13.81 ± 0.33 ^c^
Warner–Bratzler shear force (N)	11.99 ± 0.79 ^b^	16.39 ± 0.95 ^a^	15.55 ± 1.52 ^a^

K—control group; P_1_—share of hemp products in the roast: seeds 8%, flour 0.2%, linseed oil 2%; P_2_—share of hemp products in the roast: seeds 4%, flour 0.2%, linseed oil 6%, average values marked with different letters in rows differ statistically significantly: a, b, c at *p* < 0.05.

**Table 3:** Effect of enrichment with seeds, flour, and hemp oil on the basic chemical composition, fiber content, tocopherol, cholesterol, and amino acid profile of poultry roast (x ± s).

Studied Parameters	Poultry Roast		
	Group K	Group P_1_	Group P_2_
Total ash %	1.30 ± 0.10 ^a^	1.54 ± 0.17 ^b^	1.61 ± 0.25 ^b^
Protein (N × 6.25) g/100 g	21.71 ± 0.85 ^a^	23.06 ± 0.40 ^b^	22.36 ± 0.31 ^a^
Fat %	14.35 ± 0.49 ^a^	12.04 ± 0.18 ^b^	12.35 ± 1.10 ^b^
Fiber %	<0.05 ± 0.00 ^c^	2.70 ± 0.20 ^a^	1.97 ± 0.10 ^b^
Vit. E, as DL-alpha-Tocopherol acetate (mg/100 g)	0.27 ± 0.05	0.32 ± 0.06	0.33 ± 0.05
Amino acid content (g/100 g):			
Lysine	2.43 ± 0.15 ^a^	2.15 ± 0.13 ^a^	1.90 ± 0.10 ^b^
Methionine, expressed as methionine sulfone	0.46 ± 0.07	0.50 ± 0.06	0.41 ± 0.01
Cysteine, expressed as cysteic acid	0.09 ± 0.04	0.11 ± 0.08	0.08 ± 0.04
Aspartic acid	1.99 ± 0.06 ^b^	2.15 ± 0.14 ^a^	2.10 ± 0.16 ^b^
Threonine	0.97 ± 0.07	1.02 ± 0.04	0.88 ± 0.05
Serine	0.87 ± 0.06	0.99 ± 0.08	0.70 ± 0.04
Glutamic acid	3.51 ± 0.20	3.62 ± 0.12	3.10 ± 0.12
Proline	0.89 ± 0.05	0.90 ± 0.08	0.78 ± 0.04
Glycine	0.96 ± 0.11	0.98 ± 0.10	0.89 ± 0.08
Alanine	1.31 ± 0.09	1.16 ± 0.04	1.92 ± 0.05
Valine	1.07 ± 0.07	1.01 ± 0.04	0.87 ± 0.04
Isoleucine	1.08 ± 0.06	0.93 ± 0.06	0.94 ± 0.04
Leucine	1.70 ± 0.09	1.89 ± 0.05	1.83 ± 0.04
Tyrosine	0.99 ± 0.06 ^a^	0.81 ± 0.06 ^a^	0.54 ± 0.04 ^b^
Phenylalanine	0.85 ± 0.06	0.86 ± 0.02	0.73 ± 0.05
Histidine	0.49 ± 0.04	0.54 ± 0.03	0.49 ± 0.01
Arginine	0.97 ± 0.02 ^b^	1.16 ± 0.09 ^a^	1.12 ± 0.05 ^a^

K—control group; P_1_—share of hemp products in the roast: seeds 8%, flour 0.2%, linseed oil 2%; P_2_—share of hemp products in the roast: seeds 4%, flour 0.2%, linseed oil 6%, average values marked with different letters in rows differ statistically significantly: a, b, c at *p* < 0.05.

**Table 4:** Impact of enrichment with seeds, flour, and hemp oil on the fatty acid profile of poultry roast (x ± s).

Studied Parameters	Poultry Roast		
	Group K	Group P_1_	Group P_2_
Saturatet acids (SFA)			
*Decanoic acid C 10:0*	0.05 ± 0.01	<0.05	<0.05
*Lauric acids C 12:0*	0.12 ± 0.04	0.12 ± 0.03	0.12 ± 0.05
*Tetradecanoic acid C 14:0*	1.07 ± 0.05 ^a^	0.52 ± 0.03 ^b^	0.46 ± 0.04 ^b^
*Pentadecanoic acid C 15:0*	0.07 ± 0.01	0.08 ± 0.02	0.06 ± 0.02
*Heksadecanoic acid C 16:0*	24.43 ± 0.14 ^a^	17.21 ± 0.15 ^b^	16.04 ± 0.28 ^b^
*Heptadecanoic acid C 17:0*	0.23 ± 0.01 ^a^	0.13 ± 0.01 ^b^	0.12 ± 0.01 ^b^
*Octadecanoic acid C 18:0*	10.11 ± 0.20 ^a^	5.96 ± 0.15 ^b^	5.54 ± 0.08 ^b^
*Eicosanoic acid C 20:0*	0.18 ± 0.02 ^b^	0.44 ± 0.01 ^a^	0.45 ± 0.03 ^a^
*Docosanoic acid C 22:0*	0.13 ± 0.04	0.24 ± 0.08	0.26 ± 0.10
*Tetracosanoic acid C 24:0*	0.07 ± 0.02	0.11 ± 0.01	0.12 ± 0.02
Σ saturated acids (SFA)	36.46 ± 0.20 ^a^	24.81 ± 0.12 ^b^	23.17 ± 0.10 ^c^
Monosaturated fatty acids (MUFA)			
*Oleomyristic acid C 14:1*	0.06 ± 0.01	<0.1 ± 0.00	0.08 ± 0.00
*Heksadecanoic acid C 16:1*	3.28 ± 0.07 ^a^	3.00 ± 0.09 ^b^	2.85 ± 0.05 ^b^
*Heptadecenoic acid C 17:1*	0.20 ± 0.01	0.09 ± 0.01	0.09 ± 0.02
*Cis-9-octadecenoic acid C 18:1*	37.57 ± 0.30 ^a^	28.55 ± 0.40 ^b^	27.30 ± 0.42 ^b^
*Cis-11-octadecenoic acid C 18:1*	2.55 ± 0.04 ^a^	1.61 ± 0.02 ^b^	1.51 ± 0.03 ^b^
*Kwas cis-11-eikozenowy C 20:1*	0.66 ± 0.01 ^a^	0.43 ± 0.03 ^b^	0.44 ± 0.05 ^b^
*Tetracosoic acids C 24:1*	<0.05	0.06 ± 0.01	<0.05
Σ monosaturated fatty acids (MUFA)	44.32 ± 0.18 ^a^	33.74 ± 0.32 ^b^	32.27 ± 0.26 ^b^
Polyunsaturated fatty acid (PUFA)			
*Octadecadienoic acid C 18:2*	16.27 ± 0.14 ^b^	33.04 ± 0.14 ^a^	35.00 ± 0.14 ^a^
*Alpha-octadecatrienoic acid C 18:3*	1.13 ± 0.10 ^c^	6.23 ± 0.25 ^b^	7.71 ± 0.20 ^a^
*Eicosadienoic acid C 20:2*	0.40 ± 0.03 ^a^	0.18 ± 0.02 ^b^	0.16 ± 0.02 ^b^
*Eicosatrienic acid C 20:3n3*	0.08 ± 0.01	<0.05	<0.05
*Docosapentaenoic acid C 22:5n3*	0.09 ± 0.03	0.05 ± 0.02	0.05 ± 0.01
*Arachidonic acid C 20:4n6*	0.56 ± 0.11	0.67 ± 0.12	0.75 ± 0.13
*Eicosatrienic acid C 20:3n6*	0.11 ± 0.02	0.10 ± 0.01	0.10 ± 0.01
Σ polyunsaturated fatty acid	18.64 ± 0.24 ^c^	40.27 ± 0.16 ^b^	43.77 ± 0.40 ^a^
*Fatty acids N-3* g/100 g	0.20 ± 0.50 ^c^	0.86 ± 0.20 ^b^	0.98 ± 0.01 ^a^
*Fatty acids N-6* g/100 g	2.44 ± 0.28 ^b^	4.25 ± 0.60 ^a^	4.44 ± 0.30 ^a^
Cholesterol (mg/100 g)	80.64 ± 2.10 ^a^	77.89 ± 1.95 ^b^	74.02 ± 2.40 ^c^

K—control group; P_1_—share of hemp products in the roast: seeds 8%, flour 0.2%, linseed oil 2%; P_2_—share of hemp products in the roast: seeds 4%, flour 0.2%, linseed oil 6%, average values marked with different letters in rows differ statistically significantly: a, b, c at *p* < 0.05.

**Table 5:** Impact of enrichment with seeds, flour, and hemp oil on the sensory characteristics of poultry roast (points).

Studied Parameters	Poultry Roast		
	Group K	Group P_1_	Group P_2_
Smell intensity	3.80 ± 0.42	4.00 ± 0.40	4.10 ± 0.30
Taste intensity	4.00 ± 0.56	4.40 ± 0.42	4.40 ± 0.53
Smell desirability	3.80 ± 0.60 ^b^	4.64 ± 0.35 ^a^	4.60 ± 0.32 ^a^
Taste desirability	3.80 ± 0.44 ^b^	4.40 ± 0.35 ^a^	3.60 ± 0.40 ^b^
Desirability of color	4.00 ± 0.44	4.20 ± 0.35	3.80 ± 0.40
Juiciness	4.20 ± 0.50	4.00 ± 0.42	4.20 ± 0.50
Tenderness	4.30 ± 0.60 ^a^	3.60 ± 0.40 ^b^	3.80 ± 0.46 ^b^
Binding	3.60 ± 0.40 ^b^	4.40 ± 0.42 ^a^	4.00 ± 0.50 ^b^
Consistency	3.80 ± 0.32 ^b^	4.40 ± 0.46 ^a^	4.40 ± 0.46 ^a^
General acceptability	3.94 ± 0.46	4.20 ± 0.52	4.00 ± 0.36

K—control group; P_1_—share of hemp products in the roast: seeds 8%, flour 0.2%, linseed oil 2%; P_2_—share of hemp products in the roast: seeds 4%, flour 0.2%, linseed oil 6%, average values marked with different letters in rows differ statistically significantly: a, b at *p* < 0.05. Sensory scale: smell/teste intensity: 1—changed, 2—moderately changed, 3—typical, weak; 4—typical, strong, 5—typical, very strong; smell/taste desirability: 1—not desirable, 2—fairly desirable, 3—desirable, 4—very desirable, 5—highly desirable; desirability of color: 1—not desirable, 2—fairly desirable, 3—desirable, 4—very desirable, 5—highly desirable; juiciness: 1—very dry, 2—dry, 3—slightly juicy, 4—juicy, 5—very juicy; tenderness: 1—very hard, 2—hard, 3—slightly tender, 4—tender, 5—very tender; binding/consistency: 1—not desirable, 2—fairly desirable, 3—desirable, 4—very desirable, 5—highly desirable.

## Data Availability

Data is contained within the article.

## References

[1] Ursachi C.Ș., Perța-Crișan S., Munteanu F.-D. (2020). Strategies to Improve Meat Products’ Quality. Foods.

[2] Farinon B., Molinari R., Costantini L., Merendino N. (2020). The seed of industrial hemp (*Cannabis sativa* L.): Nutritional Quality and Potential Functionality for Human Health and Nutrition. Nutrients.

[3] Ravani A., Sharma H.P., Chhikara N., Panghal A., Chaudhary G. (2022). Meat Based Functional Foods. Bioprocessing in Food Science, vol. 2, Functional Foods.

[4] Beriain M.J., Gómez I., Ibáñez F.C., Sarriés M.V., Ordóñez A.I., Holban A.M., Grumezescu A.M. (2018). Chapter 1-Improvement of the functional and healthy properties of meat products. Food Quality: Balancing Health and Disease.

[5] Kerner K., Jõudu I., Tänavots A., Venskutonis P.R. (2021). Application of Raw and Defatted by Supercritical CO_2_ Hemp Seed Press-Cake and Sweet Grass Antioxidant Extract in Pork Burger Patties. Foods.

[6] Albergamo A., Vadalà R., Nava V., Bartolomeo G., Rando R., Colombo N., Gualtieri R., Petracci M., Di Bella G., Costa R. (2022). Effect of Dietary Enrichment with Flaxseed, Vitamin E and Selenium, and of Market Class on the Broiler Breast Meat—Part 1: Nutritional and Functional Traits. Nutrients.

[7] Albergamo A., Vadalà R., Metro D., Nava V., Bartolomeo G., Rando R., Macri A., Messina L., Gualtieri R., Colombo N. (2021). Physicochemical, nutritional, microbiological, and sensory qualities of chicken burgers reformulated with mediterranean plant ingredients and health-promoting compounds. Foods.

[8] Naumova N., Lukin A., Bitiutskikh K. (2017). Organoleptic evaluation of the quality of the enriched chopped semi-finished meat products. Bull. Transilv. Univ. Brasov. For. Wood Ind. Agric. Food Engineering. Ser. II.

[9] Lima T.L.S., da Costa G.F., Alves R.D.N., de Araújo C.D.L., da Silva G.F.G., Ribeiro N.L., de Figueiredo C.F.V., de Andrade R.O. (2022). Vegetable oils in emulsified meat products: A new strategy to replace animal fat. Food Sci. Technol..

[10] Botella-Martínez C., Sayas-Barberá E., Pérez-Álvarez J., Viuda-Martos M., Fernández-López J. (2022). Chia and hemp oils based gelled emulsions as replacers of pork backfat in burgers: Effect on lipid profile, technological attributes and oxidation stability during frozen storage. Int. J. Food Sci. Technol..

[11] Barros J.C., Munekata P.E.S., de Carvalho F.A.L., Pateiro M., Barba F.J., Domínguez R., Trindade M.A., Lorenzo J.M. (2020). Use of tiger nut (*Cyperus esculentus* L.) oil emulsion as animal fat replacement in beef burgers. Foods.

[12] Los P.R., Marson G.V., Dutcosky S.D., Nogueira A., Marinho M.T., Simões D.R.S. (2020). Optimization of beef patties produced with vegetable oils: A mixture design approach and sensory evaluation. Food Sci. Technol..

[13] Heck R.T., Saldaña E., Lorenzo J.M., Correa L.P., Fagundes M.B., Cichoski A.J., de Menezes C.R., Wagner R., Campagnol P.C.B. (2019). Hydrogelled emulsion from chia and linseed oils: A promising strategy to produce low-fat burgers with a healthier lipid profile. Meat Sci..

[14] Beiloune F., Bolumar T., Toepfl S., Heinz V. (2014). Fat Reduction and Replacement by Olive Oil in Bologna Type Cooked Sausage. Quality and Nutritional Aspects. Food Nutr. Sci..

[15] Hathwar S.C., Rai A.K., Modi V.K., Narayan B. (2012). Characteristics and consumer acceptance of healthier meat and meat product formulations—A review. J. Food Sci. Technol..

[16] Zahari I., Ferawati F., Helstad A., Ahlström C., Östbring K., Rayner M., Purhagen J.K. (2020). Development of High-Moisture Meat Analogues with Hemp and Soy Protein Using Extrusion Cooking. Foods.

[17] Oseyko M., Sova N., Chornei K. (2021). Substantiation of hemp seeds storage and processing technologies for functional, dietary and specialty products. Review. Ukr. Food J..

[18] Zinina O., Merenkova S., Galimov D., Okuskhanova E., Rebezov M., Khayrullin M., Anichkina O. (2020). Effects of Microbial Transglutaminase on Technological, Rheological, and Microstructural Indicators of Minced Meat with the Addition of Plant Raw Materials. Int. J. Food Sci..

[19] Gambuś H., Litwinek D., Sabat R., Wywrocka-Gurgul A., Szary-Sworst K., Baczyński J. (2020). Chemical composition and health-promoting values of seeds, oil and hemp flour (Cannabis sativa L.). Żywność a Oczekiwania Współczesnego Konsumenta.

[20] Zając M., Guzik P., Kulawik P., Tkaczewska J., Florkiewicz A., Migdał W. (2019). The quality of pork loaves with the addition of hemp seeds, de-hulled hemp seeds, hemp protein and hemp flour. LWT.

[21] Kotecka-Majchrzak K., Kasałka-Czarna N., Spychaj A., Mikołajczak B., Montowska M. (2021). The Effect of Hemp Cake (*Cannabis sativa* L.) on the Characteristics of Meatballs Stored in Refrigerated Conditions. Molecules.

[22] Dąbrowski G., Skrajda M.N. (2016). Lipid and protein fraction of hemp seed (C. sativa L.) and its beneficial impact on human health. J. Educ. Health Sport.

[23] Markowska J., Polak E., Drabent A., Żak A. (2021). Hemp *Cannabis sativa* L.-types, properties, uses. ŻYWNOŚĆ. Nauka. Technologia. Jakość.

[24] Teleszko M., Zając A., Rusak T. (2022). Hemp Seeds of the Polish ‘Bialobrzeskie’ and ‘Henola’ Varieties (*Cannabis sativa* L. var. *sativa*) as Prospective Plant Sources for Food Production. Molecules.

[25] Wang Q., Xiong Y.L. (2019). Processing, Nutrition, and Functionality of Hempseed Protein: A Review. Compr. Rev. Food Sci. Food Saf..

[26] Pojić M., Hadnađev T.D., Hadnađev M., Rakita S., Brlek T. (2015). Bread Supplementation with Hemp Seed Cake: A By-Product of Hemp Oil Processing. J. Food Qual..

[27] Amirkhanov K., Igenbayev A., Nurgazezov A., Okuskhanov E., Kassymov S., Muslimova N., Yessimbeko Z. (2017). Comparative Analysis of Red and White Turkey Meat Quality. Pak. J. Nutr..

[28] Oblakova M., Ribarski S., Oblakov N., Hristakieva P. (2016). Chemical composition and quality of turkey-broiler meat from crosses of layer light (LL) and meat heavy (MH) turkey. Trakia J. Sci..

[29] Petrescu C.A., Ciobanu M.M., Lazăr R., Boisteanu P.C., Usturoi M.G., Raju R.N. (2020). Research On the Quality of Physical Indicators of the Turkey Meat Obtained from the Big but 6 Hybrid Slaughtered at Different Age. Sci. Pap. Ser. D Anim. Sci..

[30] Augustyńska-Prejsnar A., Sokołowicz Z., Ormian M., Tobiasz-Salach R. (2022). Nutritional and Health-Promoting Value of Poultry Meatballs with the Addition of Plant Components. Foods.

[31] (1976). Commission International de Eclarage (1971) to 1-1.

[32] Augustyńska-Prejsnar A., Ormian M., Sokołowicz Z., Kačániová M. (2022). The Effect of the Addition of Hemp Seeds, Amaranth, and Golden Flaxseed on the Nutritional Value, Physical, Sensory Characteristics, and Safety of Poultry Pâté. Appl. Sci..

[33] Broune M.C. (1978). Texture profile analysis. Food Technol..

[34] Herrero A., Ordóñez J., de Avila R., Herranz B., de la Hoz L., Cambero M. (2007). Breaking strength of dry fermented sausages and their correlation with texture profile analysis (TPA) and physico-chemical characteristics. Meat Sci..

[35] Baryłko-Pikielna N., Matuszewska I. (2009). Sensory Testing of Food. Basics-Methods–Application.

[36] (2010). General Guidelines for the Design of a Sensory Analysis Laboratory.

[37] (2018). StatSoft Electronic Statistics Textbook.

[38] Bozhko N., Pasichnyi V., Tischenko V., Marynin A., Shubina Y., Strashynskyi I. (2021). Determining the nutritional value and quality indicators of meat-containing bread made with hemp seeds flour (Cannabis sativa L.). East. Eur. J. Enterp. Technol..

[39] Sun G., Xiong Y., Feng X., Fang Z. (2022). Effects of incorporation of hempseed meal on the quality attributes of chicken sausage. Futur. Foods.

[40] Yuan D., Cao C., Kong B., Sun F., Zhang H., Liu Q. (2022). Micronized cold-pressed hemp seed cake could potentially replace 50% of the phosphates in frankfurters. Meat Sci..

[41] Zając M., Świątek R. (2015). The effect of hemp seed and linseed addition on the quality of liver pâtés [pdf]. Acta Sci. Pol. Technol. Aliment..

[42] Merenkova S.P., Zinina O.V., Khayrullin M.F., Bychkova T.S., Moscow L.A. (2020). Study of the rheological properties of meat-vegetable minces. IOP Conference Series: Earth and Environmental Science.

[43] Jarosz M., Rychlik E., Stoś K., Charzewska J. (2020). Normy żywienia dla populacji Polski i ich zastosowanie. Nutrition Standards for the Polish Population and Their Application.

[44] van Vliet S., A Burd N., van Loon L.J. (2015). The Skeletal Muscle Anabolic Response to Plant- versus Animal-Based Protein Consumption. J. Nutr..

[45] Carlson J.L., Erickson J.M., Lloyd B.B., Slavin J.L. (2018). Health Effects and Sources of Prebiotic Dietary Fiber. Curr. Dev. Nutr..

[46] Jiang Q., Im S., Wagner J.G., Hernandez M.L., Peden D.B. (2021). Gamma-tocopherol, a major form of vitamin E in diets: Insights into antioxidant and anti-inflammatory effects, mechanisms, and roles in disease management. Free Radic. Biol. Med..

[47] Shahidi F., Pinaffi-Langley A.C.C., Fuentes J., Speisky H., de Camargo A.C. (2021). Vitamin E as an essential micronutrient for human health: Common, novel, and unexplored dietary sources. Free. Radic. Biol. Med..

[48] Lorenzo J.M., Domínguez R., Agregán R., Gonçalves A. (2016). Effect of fat replacement by olive oil on the physico-chemical properties, fatty acids, cholesterol and tocopherol content of pâté. Grasas y Aceites.

[49] Rodriguez-Estrada M., Penazzi G., Caboni M.F., Bertacco G., Lercker G. (1997). Effect of different cooking methods on some lipid and protein components of hamburgers. Meat Sci..

[50] Alejandre M., Passarini D., Astiasarán I., Ansorena D. (2017). The effect of low-fat beef patties formulated with a low-energy fat analogue enriched in long-chain polyunsaturated fatty acids on lipid oxidation and sensory attributes. Meat Sci..

[51] Selani M.M., Shirado G.A., Margiotta G.B., Saldaña E., Spada F.P., Piedade S.M., Contreras-Castillo C.J., Canniatti-Brazaca S.G. (2016). Effects of pineapple byproduct and canola oil as fat replacers on physicochemical and sensory qualities of low-fat beef burger. Meat Sci..

[52] Lorenzo J.M., Munekata P.E.S., Pateiro M., Campagnol P.C.B., Domínguez R. (2016). Healthy Spanish salchichón enriched with encapsulated n − 3 long chain fatty acids in konjac glucomannan matrix. Food Res. Int..

[53] Chen J., Liu H. (2020). Nutritional Indices for Assessing Fatty Acids: A Mini-Review. Int. J. Mol. Sci..

[54] Rustan A.C., A Drevon C. (2005). Fatty Acids: Structures and Properties. eLS.

[55] De Carvalho C.C.C.R., Caramujo M.J. (2018). The Various Roles of Fatty Acids. Molecules.

[56] Churchward C.P., Alany R.G., Snyder L.A.S. (2018). Alternative antimicrobials: The properties of fatty acids and monoglycerides. Crit. Rev. Microbiol..

[57] Użarowska M., Surman M., Janik M. (2018). Two faces of cholesterol: Physiological importance and role in disease pathogenesis. Space.

